# GLR Channels Are Involved in the Mechanism of Chloroplast Avoidance Response in *Lemna trisulca*

**DOI:** 10.3390/plants14192990

**Published:** 2025-09-27

**Authors:** Weronika Krzeszowiec, Halina Gabryś

**Affiliations:** Department of Plant Biotechnology, Faculty of Biochemistry, Biophysics and Biotechnology, Jagiellonian University in Kraków, Gronostajowa 7, 30-387 Kraków, Poland; halina.gabrys@uj.edu.pl

**Keywords:** chloroplast movements, GLR channels, *Lemna trisulca* (duckweed), MK-801 (dizocilpine), phototropin 2

## Abstract

(1) Background: The complete signalling pathway leading to light-induced chloroplast movement in plant cells is not yet fully understood. The process may involve GLR channels, which have previously been shown to participate in light signalling in plants. Therefore, using in vivo photometry we analysed chloroplast movements in the water plant *Lemna trisulca* treated with GLR channel inhibitors, MK-801 and CNQX. (2) Results: MK-801, a non-competitive antagonist that blocks NMDA channels was found to inhibit the avoidance response of chloroplasts controlled by phototropin2. This inhibition depends on pH and requires alkaline conditions. On the contrary, CNQX, a competitive receptor antagonist that blocks AMPA channels did not change the parameters of chloroplast movements in either mild alkaline or acidic conditions. (3) Conclusions: Our study reveals that GLR NMDA channels play a role in chloroplast movements and provides new insights into the phot2 signalling pathway. The results also suggest that the activity of these channels depends on pH, similar to NMDA receptors present in animal cells.

## 1. Introduction

GLutamate Receptor-like (GLR) channels are multimeric transmembrane structures in plant cells that operate as nonselective, ionotropic, ligand-gated receptors [1,2]. They are homologous to ionotropic Glutamate Receptors (iGluRs), which play a key role in neurotransmission in animals [3]. There are three types of animal iGluR, namely, NMDA, AMPA and kainate receptors. These receptors are named after the agonists that activate them: NMDA (N-methyl-d-aspartate), AMPA (α-amino-3-hydroxyl-5-methyl-4-isoxazole-propionate) and kainic acid. In animals, the gating mechanism depends on the binding of glutamate and glycine. In contrast, the gating principles have not been established in plant cells [4].

The growing number of studies published concerning GLRs comes from their role in a variety of vital physiological and developmental processes, e.g., seed germination, pollen tube growth, reproduction and chemotaxis, regulation of stomatal aperture, ion transport, long-distance signalling, responses to salt stress, wounding, and pathogens [4,5,6]. Although GLR channels have been known in plants for over twenty years [1], the list of processes in which they are involved has not been finalised. In addition, the mechanisms of their opening and closing have not been discovered, and the composition of individual channels, as well as the dependence of the channel structure on their ligand specificity, have not been found. Furthermore, it has not been determined which ions are transported through GLRs under specific conditions. In summary, current research on the cellular functioning of these channels is still in its early stages.

Our studies focus on deciphering the role of GLRs in light signal transduction pathways. In previous papers, we demonstrated the expression patterns of GLR genes as affected by blue and red light photoreceptors in mature Arabidopsis leaves [7], and the involvement of the channels in the photomorphogenesis of Arabidopsis seedlings [8]. The present research is intended to determine the role of GLR channels in chloroplast movements: fast physiological plant cell responses to light.

Chloroplast movements belong to the basic processes controlled by blue light in plants. These movements commonly occur throughout the plant kingdom. They have been found to be very slow or absent only in a few species [9]. In low light, chloroplasts accumulate in the cell at better-illuminated walls. On the contrary, excess light causes an avoidance response, with chloroplasts taking up positions along less illuminated walls [10,11,12]. The accumulation response optimises photosynthesis while the avoidance response is considered to protect the photosynthetic apparatus, although the latter opinion has recently been questioned [13]. Two photoreceptors, phototropin 1 and 2, control chloroplast movements in vascular plants. The motile system involved in chloroplast movement is actin and possibly myosin [14,15]. The signalling pathways from phototropins to the motile system include several proteins that interact with actin, while phosphoinositides and Ca^2+^ play the role of secondary messengers [16]. Surprisingly, after several decades of research, these signal transmission pathways are only partially understood.

The regulatory role of Ca^2+^ in the chloroplast movements of *Lemna trisulca* was first studied by Tlałka and Gabryś [17]. The authors concluded that the calcium necessary for chloroplast responses is derived from intracellular stores. Also Mg^2+^ was shown to be involved in restoring movements after chelating calcium ions with EGTA. Taking into account the established role of GLRs as Ca^2+^ channels [4], we hypothesised that they participate in the regulation of chloroplast responses to blue light. To verify this hypothesis, we used specific inhibitors known from animal studies of iGluRs.

Inhibitors are frequently used in research into iGluR channels. Moreover, many of them have been discovered and described for their potential treatment of pain, schizophrenia and stroke in humans. Specific iGLuR inhibitors have also been used in plant studies, listed here in order of publication date of their first use: DNQX [1], CNQX and MNQX [18], MK-801 and NBQX [19,20], AP-5 [20], and AP-7 and kynurenic acid [21]. The use of glutamate receptor inhibitors raises the question of pH conditions in experiments involving plant cells. The optimum pH for growth of most animal cells ranges from 7.2 to 7.4 [22]. On the contrary, plant media predominantly have acidic pH. In consequence, most results on GLR channel inhibition in plants were obtained at low pH values. The functioning of animal iGLURs appears to be stimulated in alkaline conditions. Therefore, we examined whether mildly alkaline pH affects the effectiveness of GLR inhibitors for chloroplast movements.

## 2. Results

**Figure 1:**
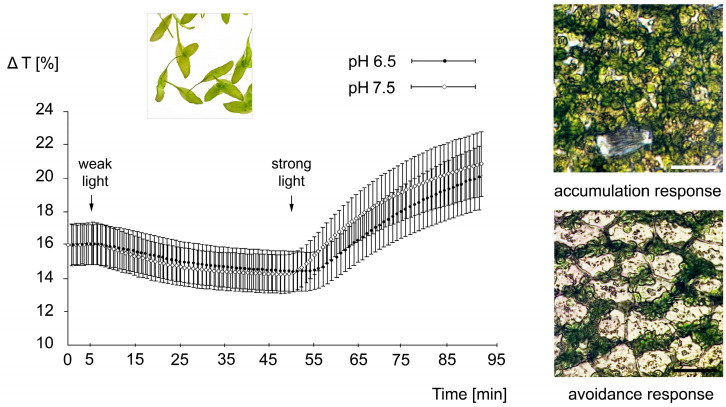
The impact of pH on chloroplast movement responses to light in the aquatic angiosperm, *Lemna trisulca*. The responses were activated by weak (1.4 μmol m^–2^ s^–1^) followed by strong (108 μmol m^–2^ s^–1^) continuous blue light. Transmittance changes obtained at pH 6.5 and pH 7.5, representing the means of 26–30 individual measurements are depicted. Arrows indicate the start of irradiation with weak and strong blue light. Inset: Lemna fronds. Microphotographs: chloroplast distribution after 1 h irradiation with weak blue light (saturated accumulation response) or with strong blue light (saturated avoidance response). Bars represent 50 µm.

To answer the question as to whether GLR channels participate in chloroplast movements, we used the aquatic angiosperm *Lemna trisulca*. This species has a unique morphology, with submerged fronds composed of 1–3 cell layers. Thus, it is an excellent model for chloroplast movement assays, suitable for both photometric measurements and simple microscopic examination (Figure 1). Furthermore, the anatomy of *L. trisulca* facilitates the application of inhibitors. For land plants, the delivery of chemicals to the mesophyll requires infiltration, i.e., the removal of air from intercellular spaces and filling them with the experimental solution. The degree of infiltration is difficult to evaluate, particularly as the procedure must be performed in very week green safe light. Moreover, the complete removal of air, which guarantees full access of inhibitors to mesophyll cells, may cause mechanical or osmotic stress.

### 2.1. No Effect of pH on Chloroplast Movements

To evaluate chloroplast responses we measured two parameters: transmittance changes accompanying accumulation/avoidance in weak/strong blue light and the respective velocities. Chloroplast movements were compared in the samples treated with 20 mM PIPES at pH 6.5 and 7.5. The plots in Figure 1 show no difference in the responses at different pH values. The difference between average amplitudes calculated for pH 6.5 and 7.5 is as low as 2% for the accumulation response and slightly less than 5% for the avoidance response. Each plot is an average of 30 experiments. The microphotographs next to the plots show the stationary position of the chloroplasts in weak and strong blue light.

### 2.2. Effect of GLR Inhibitors at pH 6.5 and pH 7.5 on Chloroplast Movements

**Figure 2:**
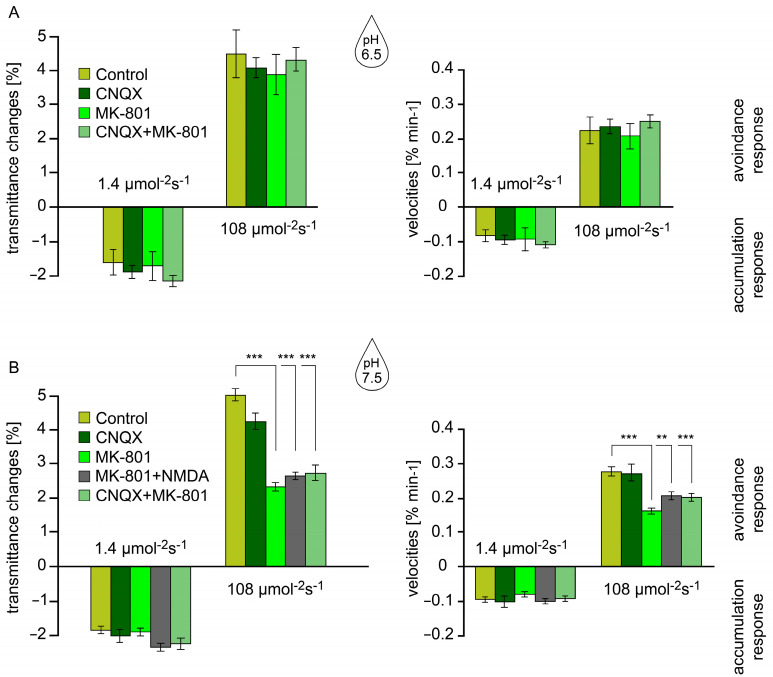
The impact of GLR channel inhibitors on chloroplast movement responses to light in the aquatic angiosperm, *Lemna trisulca*. The responses were activated by weak (1.4 μmol m^–2^ s^–1^) followed by strong (108 μmol m^–2^ s^–1^) continuous blue light. Bar charts demonstrate the amplitudes and velocities of transmittance changes reflecting chloroplast positions after 45 min of continuous blue light at pH 6.5 (**A**) and pH 7.5 (**B**). The results are based on the means of at least 12 experiments, and error bars reflect standard errors. Asterisks indicate the statistical significance of the difference: ** *p* ≤ 0.01, *** *p* ≤ 0.001.

Two inhibitors were used to establish whether specific GLRs are involved in chloroplast movements. To answer this question, we used MK-801 (dizocilpine), a non-competitive antagonist, that blocks NMDA channels and CNQX (6-cyano-7-nitroquinoxaline-2,3-dione), a competitive receptor antagonist, that blocks AMPA channels. In the first set of experiments we tested the effects of both inhibitors at a pH of 6.5, commonly used in plant studies. Neither inhibitor affected chloroplast redistribution at this pH value (Figure 2A).

On the contrary, a significant inhibitory effect was seen when tentative GLR NMDA channels were blocked with MK-801 at pH 7.5 (Figure 2B). The chloroplast response to strong light, i.e., the avoidance response, was specifically inhibited. Compared to the controls, the average amplitudes were reduced to 46% and velocities dropped to 56%. The accumulation response in weak light, which is known to have a different mechanism, was undisturbed.

CNQX did not change chloroplast response parameters at pH 7.5, similar to the result obtained at the lower pH value (Figure 2). Chloroplast movements were also tested at pH 7.5 in the presence of both inhibitors, MK-801 and CNQX, to investigate whether both channel types cooperate in signal transduction. Here, the inhibition was similar to that with MK-801 used alone, suggesting that only tentative GLR NMDA channels are involved (Figure 2B). Altogether, our results suggest that GLR AMPA channels do not participate in chloroplast responses to blue light.

An attempt was made to reverse the inhibition caused by MK-801 by introducing NMDA (N-methyl-D-aspartate), an agonist of these channels (Figure 2B). Unfortunately, our findings were inconclusive; some experimental series showed reversibility, while in other series the agonist was inactive. Further experiments are necessary to clarify this discrepancy.

## 3. Discussion

Overall, blue-light induced chloroplast movements in *Lemna trisulca* show close similarities to those in the *Arabidopsis thaliana* model plant [17]. To verify this observation at the molecular level, mutants in *L. trisulca* are necessary, but the transformation protocol for this duckweed species is still missing.

As discussed above, iGluR inhibitors are commonly used in plant research. Questions arise as to whether they are specific to plant GLRs and stable in plant tissue. The specificity of MK-801 and CNQX has been confirmed many times in animal tissues. MK-801 is a non-competitive inhibitor of NMDA channels in mammalian cells which selectively antagonises the effects of NMDA by blocking NMDA channels, but not kainate and AMPA channels [23]. The only additional effect found for MK-801, since its discovery in 1982, is the blocking of nicotine receptors [24,25]. Recently, X-ray crystallography proved that MK-801 binds to NMDA receptors in an open state, thereby blocking the influx of ions [26]. The second inhibitor tested, CNQX, a member of the quinoxalinedione family, is the most widely used competitive antagonist of AMPA- and kainite-type iGLuRs [27]. However, its inhibitory properties in a neuronal setting are sometimes questioned [28].

So far, nobody has evaluated the specificity of both inhibitors in plants. Their stability in aqueous solutions was demonstrated in our previous study [8], suggesting that the inhibitors should be active in hydrated plant tissues or when applied onto aquatic species, such as *L. trisulca*.

Chloroplast movements of *Lemna trisulca* in strong blue light are reduced by half upon the addition of MK-801 in PIPES pH 7.5. This points to the involvement of tentative NMDA channels in the avoidance response. The question arises as to why MK-801 does not reduce the chloroplast response in acidic medium.

The effect of pH on MK-801 (older name dizocilpine) has been described in the animal literature. Firstly, the binding of the inhibitor and its impact on the calcium wave in cultured forebrain neurons were examined using tritium-labelled MK-801 [29]. Increasing the pH of the externally administered solution from 6.5 to 7.4 and 8.0 significantly increased the rate of association and dissociation of [3H]-dizocilpine. The level of the NMDA channel inhibition did not depend on pH. Secondly, Chen et al. [30] showed the influence of external and internal pH on NMDA receptor activation in Xenopus neuromuscular cultures in which NMDA responses were inhibited by external acidification and enhanced by intracellular acidification. Importantly, external alkalisation enhanced NMDA action, while intracellular alkalisation had no significant effect. Finally, Traynelis et al. [31] showed that NMDA channels were reversibly inhibited by extracellular protons in rat cerebellar granule neurons, as measured with whole-cell and single-channel patch-clamp. NMDA receptors are sensitive to changes in H+ within the physiological range. About half of NMDA channels are active at the physiological pH of extracellular fluid. This means that the cells can markedly influence the channel activity by slight changes in extracellular pH. Excessive activation of NMDA receptors in the central nervous system causes neuronal injury [32]. Therefore pH appears to be one of the factors that enable the animal cell to control NMDA receptor functioning and to support its viability. Our results are the first demonstration that GLR activity is regulated by a similar mechanism.

Neglecting the relationship between pH and the activity of GLR channels may be the reason for many ambiguous results that have previously been published. Conclusions are drawn regardless of the differences in pH used in the experiments; moreover, some publications do not mention pH conditions. For example, in Tapken et al. [33] electrophysiological investigations of Arabidopsis seedlings were performed at pH 6.0, and they were continued with the channel heterologously expressed in Xenopus at pH 7.2. In the studies by Qi et al. [34], an agonist stopped acting in Arabidopsis seedlings upon a pH change from 5.7 to 7.7. This fact was simply interpreted as a result of H+-amino acid symport.

Our research focuses on the potential functions of GLRs in light signalling pathways. We gather information on these channels to image their function in plants. Gene expression studies have revealed only a slight effect of light on the expression of GLR genes in Arabidopsis. In particular, phototropins did not seem to mediate this effect, because out of the 13 GLR genes tested, only the expression of AtGLR1.1 was reduced in the double mutant phot1/phot2 [7].

Research on light-regulated Arabidopsis seedling growth supports the conclusion that plants contain both types of amino acid-gated receptors, GLR-AMPA channels and GLR-NMDA channels [8]. Simultaneous application of both inhibitors, MK-801 and CNQX, showed synergic effects, which proves the interaction of both channel classes. The strongest inhibition was observed in the case of root growth. Overall, CNQX blocked the seedling growth much more strongly than MK-801. We also tested the impact of inhibitors on calcium waves. MK-801 strongly reduced the Ca^2+^ wave, while CNQX showed no significant effect.

Even though pH does not directly affect blue-light-induced chloroplast movements, the reported MK-801 inhibition of the avoidance response allows GLR NMDA channels to be added to the signal transduction pathway from phototropin2. Recently, Łabuz et al. [35] reviewed in detail the current understanding of phototropins and their role in chloroplast movements. These movements start from the light activation of two homologous photoreceptors: phot1 and phot2. While the accumulation response is directed by both photoreceptors, the avoidance response is controlled exclusively by phot2 [36]; phot1 was shown to initiate only a transient avoidance response under strong irradiation [37].

Two interesting questions posed by Łabuz et al. [35] concern the as yet unknown difference in the signalling pathways originating from phot1 and phot2, (1) why is phot1 unable to complete the chloroplast avoidance response? (2) how does phot1 determine whether the chloroplasts will perform an accumulation or avoidance? We propose that one of the missing elements in the signal transduction pathway from phot2 might be a GLR NMDA channel or channels. The findings and their implications should be discussed in the broadest context possible. Future research directions may also be highlighted.

## 4. Materials and Methods

### 4.1. Plant Material and Growth Conditions

The aquatic monocotyledonous angiosperm *Lemna trisulca* L. from the collection of the Jagiellonian University Botanical Garden in Kraków, Poland was cultured in daylight in Erlenmayer flasks kept in a north-facing window. A twice-diluted liquid medium proposed by Appentroth et al. [38] was used, i.e., 30 µM KH_2_PO_4_, 0.5 µM Ca(NO_3_)_2_, 4 mM KNO_3_, 0.5 mM MgSO_4_, 2.5 µM H_3_BO_3_, 7.5 µM, 7.5 µM MnCI_2_, 0.2 µM Na_2_MoO_4_, 12.5 µM FeEDTA. One week before the experiments, the plants were transferred to a growth chamber (Sanyo MLR-350H, Osaka, Japan) at a photoperiod of 14L/10D and a temperature of 23  ±  2 °C. They were illuminated with fluorescent lamps (Philips Master TL-D 36 W/840, Poland, Osram L36 W/77 Fluora, Germany) with an average PPFD of 110 μmol m^−2^ s^−1^. The plants were moved to a fresh medium before being transferred to the Sanyo chamber.

### 4.2. Solutions

One hour before the measurement, the *Lemna trisulca* fronds were incubated in one of the following solutions. As control solutions, 20 mM PIPES pH 6.5 (KOH) or pH 7.5 (KOH) supplemented with 0.5% DMSO were used. For inhibition of NMDA-like channels we used: 0.5 mM MK-801 in 20 mM PIPES pH 6.5 or pH 7.5 and for inhibition of AMPA-like channels: 0.5 mM CNQX in 20 mM PIPES pH 6.5 or pH 7.5. Simultaneous inhibition of both types of channels was tested with 0.5 mM MK-801 + 0.5 mM CNQX in 20 mM PIPES pH 6.5 or pH 7.5, and the reversal of inhibition with: 0.5 mM MK-801 + 0.5 mM NMDA in 20 mM PIPES pH 7.5. Stock solutions of inhibitors were prepared in DMSO. The final concentration of DMSO was 0.5% in all cases. The detailed methodology of using MK-801 and CNQX in plant studies is given in [8].

### 4.3. Photometric Method—Chloroplast Movement Measurements

A custom-made double-beam photometer [39] was used to quantitatively assess the chloroplast movements. The method is based on the fact that chloroplast redistribution changes light transmittance through the leaf. The measuring beam (monochromatic red light λ = 660 nm, 0.1 μmol m^−2^ s^−1^, frequency of 800 Hz) does not influence chloroplast movement in *L. trisulca*. Chloroplast responses were elicited by actinic light of 460 nm (Luxeon Royal Blue LXHL-FR5C diode, Philips Lumiled Lighting Comp., San Jose, CA, USA). 12 h before measurements the plants were dark-adapted in commercial drinking water (Eden Springs Pvt. Ltd., Krzeszowice, Poland; water pH 7.7; cations: Ca^2+^ 58.9 mg/L, Mg^2+^ 30.90 mg/L, Na^+^ 3.22 mg/L, K^+^ 1.27 mg/L; anions: HCO_3_^−^ 312.00 mg/L, F^−^ < 0.20 mg/L, Cl^−^ 3.50 mg/L, SO_4_^2−^ 24.00 mg/L; total minerals 448.46 mg/L) to minimise the potential impact of pH change in the Lemna growth medium. For the final hour of dark adaptation, each sample was treated with one of the solutions listed in Section 4.2. The sample thus prepared was placed on a double-cavity microscope slide and covered with a gas-permeable membrane stretched over a rubber ring. The slide was mounted in the measuring chamber using a special holder. Two concentric light beams, actinic (blue) and measuring (red) light, were delivered perpendicular to the dorsal surface of the frond. For further details on the method see Gabryś et al. [39].

After determining the initial transmittance level, the Lemna fronds were illuminated with weak blue light (1.6 μmol m^−2^ s^−1^) for 45 min, which elicited a full accumulation response of chloroplasts. This was followed by 45 min strong blue light (108 μmol m^−2^ s^−1^) saturating the avoidance response. Two parameters were calculated for both responses: amplitude—the transmittance change after 45 min, and velocity—the first derivative of the initial linear fragment of the transmittance change.

### 4.4. Statistical Analysis

The data are presented as means with standard error. A one-way analysis of variance (ANOVA) was performed, followed by a post hoc Tukey–Kramer test (multiple comparisons of means) to determine the differences between the means. The statistical analyses were performed with GraphPad InStat version 3.10. Calculations were made on the basis of 3 replicates of at least 12 samples each. In the experiments intended to gain an understanding of the mechanism of GLR action, performed at pH 7.5 (control, MK-801, MK-801 + CNQX, MK-801 + NMDA), at least double the number of samples were measured.

## 5. Conclusions

We have shown that NMDA GLR channels participate in the control of the chloroplast avoidance response in *Lemna trisulca*. As the avoidance response is activated only by strong blue light, this indicates that GLR channels are part of the phototropin2 signalling pathway.

We have also shown that GLR NMDA channels are pH-dependent, similar to NMDA receptors present in animal cells. This part of the results provides new insights into the mechanism of the action of the GLR channels in plants.

Moreover, in our study, GLR AMPA channels, which were previously shown to take part in seedling growth, proved inactive in chloroplast movements. Thus, different NMDA and AMPA-dependent GLR channels do not act redundantly, but have specialised functions in plants.

## Data Availability

The experimental data that support the findings of this study are available in the RODBUK Cracow Open Research Data Repository with the identifier https://doi.org/10.57903/UJ/PWW9HF.

## Abbreviations

The following abbreviations are used in this manuscript:

GLR	GLutamate Receptor-like channels
iGLuRs	ionotropic Glutamate Receptors
NMDA	N-methyl-D-aspartate (receptor/channel)
AMPA	α-amino-3-hydroxy-5-methyl-4-isoxazole-proprionic acid (receptor/channel)
